# Chinese Herbal Medicine Versus Placebo for the Treatment Of Chronic Obstructive Pulmonary Disease: A Protocol of Systematic Review and Meta-analysis

**DOI:** 10.1097/MD.0000000000017002

**Published:** 2019-08-30

**Authors:** Chan Xiong, Yu Li, Yan Zeng, Hua Wei, Guang-tong Zhuang, Lin Li, Li-hong Zhao, Chen-yi Li, Er-qi Qin, Juan-juan Fu

**Affiliations:** a Department of Respiratory; b Department of Cardiovascular; c Department of Research, No.3 Affiliated Hospital of Chengdu University of TCM (West District), Chengdu Pidu District Hospital of TCM; d Department of Integrated Traditional and Western Medicine, West China Hospital, Sichuan University, Chengdu, Sichuan, China.

**Keywords:** Chinese herbal medicine, chronic obstructive pulmonary disease, meta-analysis, placebo, protocol, systematic review

## Abstract

**Background:**

Chinese herbal medicine (CHM) has been shown to be effective in the treatment of stable chronic obstructive pulmonary disease (COPD) by published meta-analyses. However, disease outcomes were inconsistent and heterogeneity was observed attributed to placebo-controlled studies. We present a protocol for a systematic review aiming to evaluate the clinical efficacy and safety of CHM comparing to placebo in the treatment of stable COPD, to provide robust evidence for the use of CHM in COPD.

**Methods:**

We will comprehensively search the following 9 databases from inception to March 2019: Web of Science, PubMed, EMBASE, Cochrane Central Register of Controlled Trials (CENTRAL), Chinese National Knowledge Infrastructure (CNKI), WANFANG Database, Chinese Scientific and Technological Periodical Database (VIP) and Chinese Biomedical Database (CBM), and the Cochrane Library database. All clinical randomized controlled trials comparing CHM to placebo for the treatment of stable COPD in English or Chinese will be included. The primary outcome will be quality of life, symptom score and exacerbation frequency, and the secondary outcomes include traditional Chinese medicine syndrome score and effective rate, lung function, 6-minute walk distance, and adverse events. Data extraction and quality assessment will be performed independently by 2 reviewers. Data synthesis and risk of bias will be assessed using the Review Manager software. This protocol will be conducted according to the Preferred Reporting Item for Systematic Review and Meta-analysis Protocols (PRISMA-P) guidance.

**Results:**

This systematic review and meta-analysis will provide a high-quality comprehensive evaluation of the efficacy and safety based on current literature evidence of CHM intervention for stable COPD.

**Conclusion:**

The conclusion of this study will present the evidence of whether CHM is an effective and safe intervention for stable COPD patients.

## 1 Introduction

Chronic obstructive pulmonary disease (COPD) is a worldwide public health challenge, characterized by progressive airflow limitation owing to chronic inflammation of the airway,^[1]^ which seriously affects the quality of life of patients and has a high mortality rate. It is currently the fourth leading cause of death in the world,^[1]^ and a recent study in Lancet has identified that there are approximately 100 million patients with COPD in China^[2]^ bringing huge social and economic burden.^[3]^

The treatment of COPD has made great progress during the last decade. The main pharmacotherapies of COPD in modern medicine include inhaled bronchodilators (beta2-agonists and anticholinergics) and glucocorticoids, which has been demonstrated to be effective in relieving symptoms and reducing exacerbation risk.^[4,5]^ However, the treatment of COPD is still challenging, as the symptoms and airway inflammation can be persistent and in patients with COPD, and certain patients will continue to suffer frequent exacerbations with continuously deterioration in functional status despite aggressive medical maintenance therapy.^[6]^

Chinese herbal medicine (CHM) has been used to treat symptoms of COPD including chronic cough, dyspnea, and sputum production for thousands of years and has gradually gained popularity worldwide. COPD has been discussed and summarized as lung distention (Fei Zhang) according to traditional Chinese medicine (TCM) theory.^[7]^ CHM alone or integrated with routine pharmacologic therapy has been widely used for the treatment of COPD in China,^[8]^ and has been considered promising for improving symptoms, physiological impairment, and relieving comorbidities.^[9–11]^ A number of clinical trials^[8,12,13]^have shown the clinical efficacy of CHM in the treatment of stable COPD, which is associated with the reduction in exacerbation frequency, relieving of clinical symptoms, and improvement of pulmonary function and quality of life. However, because of the difference in the study design, included studies, and outcome measurements of these clinical trials, the results were inconsistent; therefore, meta-analyses^[14–16]^ addressing the clinical efficacy of CHM on COPD yielded variable results, which hinders the explanation of study results and subsequently its clinical use.

Importantly, with the standardization of clinical study on CHM, quality of clinical trials has been improved and an increasing number of CHM studies follows the principles of randomization, blindness, and control in recent years, of which the use of placebo is an important principle linking the implementation of ‘blindness.^[17]^ The use of placebo can exclude the placebo effect, reflecting the real efficacy and safety of the drug to the greatest extent.^[18]^ Placebo has been reported to improve subjective and objective disease outcomes in up to 30% to 40% of patients with a wide range of clinical conditions, such as pain and asthma, among others.^[19,20]^ A systematic review showed that 85% of cough relief was related to placebo treatment, and only 15% was attributed to active ingredient treatment of antitussive drugs.^[21]^ The use of placebo control in clinical trials of CHM reflects the real efficacy of the tested drugs, thus ensures the scientificity and reliability of clinical trials, which is conducive to the recognition and application of CHM worldwide.

We reviewed the published systematic reviews and meta-analyses of CHM on stable COPD. As shown in Table 1,^[14–16,22–39]^ placebo-controlled clinical trials included in these meta-analyses varied with some studied overlapped yet there was no meta-analysis that included all published placebo-controlled trials.^[15,16,22,27,30,38]^ In addition, several new published placebo-controlled trials were not included in these meta-analyses.^[9,12,40]^ Moreover, results of these meta-analyses were inconstant such as in reducing acute exacerbation and improving lung function.^[15,16,30,38,39]^ More importantly, we found that heterogeneity exists among included studies because of the difference in control groups. In a meta-analysis that included 37 randomized controlled trials (RCTs) with only 6 placebo-control RCTs, the effects of CHM on improving quality of life and 6-minute walk distance (6MWD) were significant different between studies with and without placebo controls.^[30]^

**Table 1:**
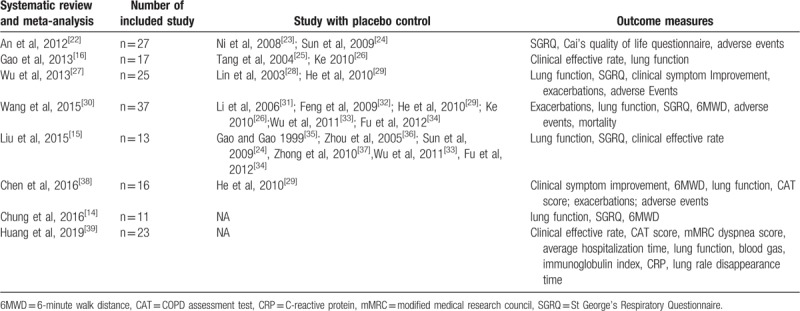
Summary of systematic reviews and meta-analyses of Chinese herbal medicine on the treatment of stable chronic obstructive pulmonary disease.

Another meta-analysis showed high heterogeneity in the outcome in terms of lung function, whereas heterogeneity was significantly reduced when placebo-controlled clinical trial was removed in the subgroup analysis,^[38]^ indicating the high heterogeneity generated by the difference in placebo control. Therefore, heterogeneity in included RCTs owing to the control arms limits the rigorism of the meta-analysis and conclusion needs to be drawn with caution, which requires further investigation. It is indispensable to further explore the effects of CHM on stable COPD by restricting the inclusion criteria for placebo-controlled RCTs.

There has been no study that has investigated the clinical efficacy of CHM for the treatment of stable COPD compared with placebo so far. We present here a protocol to comprehensively collect and sort out clinical randomized trials using CHM with placebo controls for the treatment of stable COPD, and to systematically evaluate its clinical efficacy and safety, to provide evidence-based recommendations for the use of CHM in COPD.

## 2 Methods

### 2.1 Registration

The protocol of this study has been registered on international prospective register of systematic review (PROSPERO) (registration number: CRD42019129269). This systematic review protocol will be conducted according to the Preferred Reporting Item for Systematic Review and Meta-analysis Protocols (PRISMA-P) guidance.^[41]^

### 2.2 Criteria for including studies in this review

#### 2.2.1 Types of studies

We will include all RCTs in treating stable COPD using CHM based on basic western medicine and comparing with placebo. Non-RCTs and uncontrolled clinical trials will be excluded.

#### 2.2.2 Participants

- Patients must be aged at least 18 years, regardless of their sex, race, education, or economic status.
- Patients with COPD are at stable stage, and the diagnosis of COPD should refer to the standard diagnostic criteria including the Global Initiative for Chronic Obstructive Lung Disease (GOLD)^[1]^ and the TCM diagnostic standards for COPD (diagnostic criteria are the same as GOLD).^[7]^ Stable COPD is defined that the symptoms such as cough, expectoration, and dyspnea are stable or mild.

#### 2.2.3 Exclusion criteria

- Combined with other intervention of traditional Chinese medicine, such as moxibustion, acupuncture, among others.
- Repeatedly published literature.
- Patients with significant diseases other than COPD, including a diagnosis of asthma, bronchiectasis, congestive heart failure, tuberculosis, and diffuse bronchiolitis, as well as patients with severe complications and complications of other organs.
- Literature that is abstracted without full text or lacking original data.

#### 2.2.4 Interventions and comparators

The treatment group will be administrated with CHM including decoction, granule, powder, tablet, pill, oral liquid, and paste based on routine treatment of western medicine. The control group will be treated with placebo with routine western medicine. All the medications must be administered orally. There was no limitation on specific doses and treatment duration of the treatments.

#### 2.2.5 Types of outcome measures

The primary outcomes include:

1. Quality of life: measured by a validated questionnaire, for example, St George's Respiratory Questionnaire^[42]^;
2. Symptom score: COPD assessment test^[43]^;
3. Exacerbations^[1]^: frequency of exacerbations, time to first exacerbation.

The secondary outcomes include:

1. TCM syndrome score and effective rate^[44]^;
2. Lung function^[45]^: change in forced expiratory volume in 1 second (FEV_1_);
3. 6-minute walk distance^[46]^;
4. Adverse events (any, including nonspecific adverse events, and discontinuation of CHM treatment owing to adverse events).

### 2.3 Search strategy

RCTs will be searched in the following electronic databases from their respective inception to March 2019: Web of Science, PubMed, EMBASE, Cochrane Central Register of Controlled Trials (CENTRAL), Chinese National Knowledge Infrastructure (CNKI), WANFANG Database, Chinese Scientific and Technological Periodical Database (VIP) and Chinese Biomedical Database (CBM), and the Cochrane Library database. Ambiguous literature will be manually searched to avoid missing eligible trials. Ongoing registered clinical trials will be searched on the websites of Chinese clinical trial registry (http://www.chictr.org.cn/) and international clinical trial registry (http://clinicaltrials.gov/). Additional trials will be searched by reviewing the reference lists of the retrieved articles, conference proceedings, and gray literature. The searching languages include Chinese and English. The search terms or key words will be used alone or in varying combinations. The search strategy for PubMed is shown in Table 2.

**Table 2:**
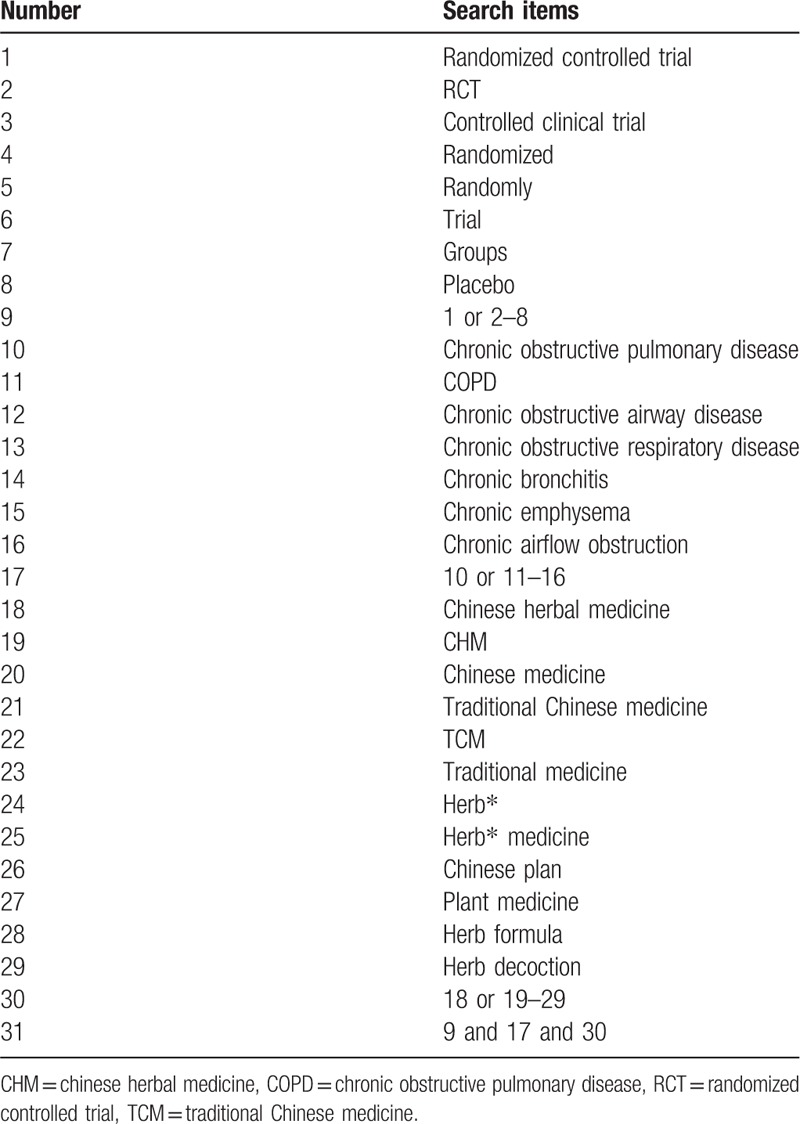
Search strategy used in PubMed database.

### 2.4 Study selection and data extraction

**Figure 1:**
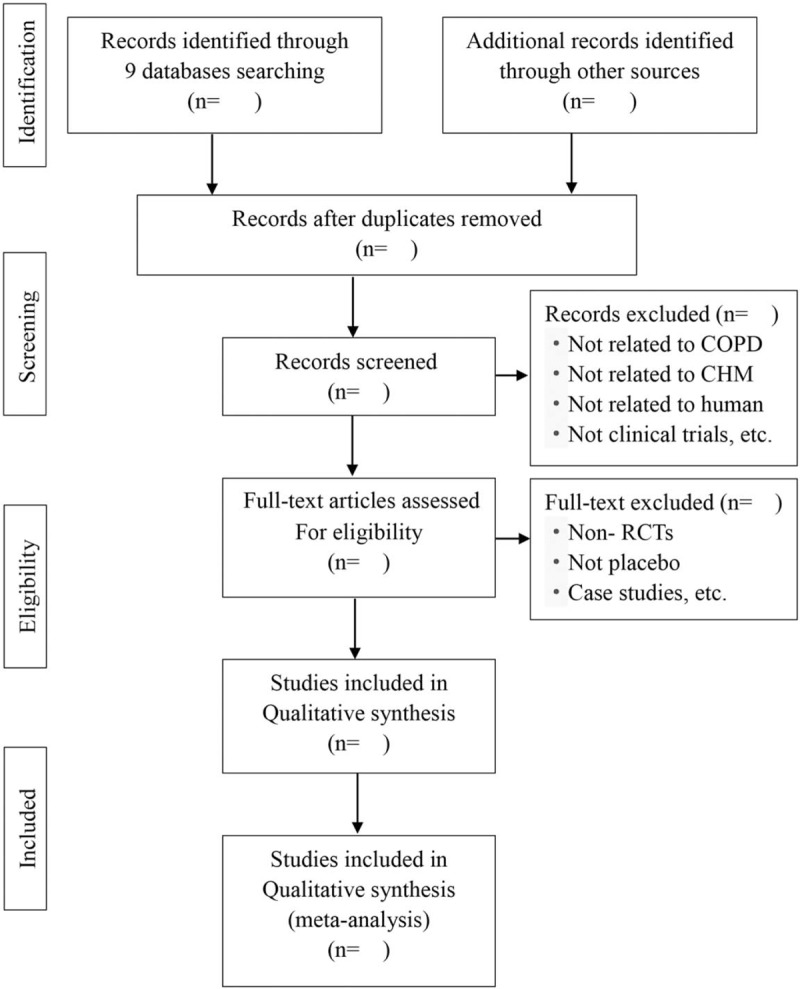
Flow diagram of the trial selection process. CHM = Chinese herbal medicine, COPD = chronic obstructive pulmonary disease, RCTs = randomized controlled trials.

Literature-retrieved citations will be managed by EndNote X9 (Thomson Reuters). Two independent reviewers (L-hZ and C-yL) will assess the title and abstract of the literature after removing duplications. The further screening will be performed to select eligible articles by reviewing the full text. Any disagreement will be resolved through further discussion with a third reviewer (J-jF). The selection process will be documented and summarized in a PRISMA flow chart (Fig. 1).

Standardized database tables will be designed for data extraction, which include title, first author, publication year, country, sample size, age and sex of the participants, intervention, treatment duration, follow-up period, outcomes, and adverse events. Data extraction will be performed independently by 2 reviewers (CX and YL), and all extracted data will be cross-checked by the 2 reviewers to ensure accuracy. If necessary, a third reviewer (Juan-juan Fu) will make the adjudication.

### 2.5 Risk of bias assessment

Quality assessment will be performed using the tool for “risk of bias” from the Cochrane Handbook for Systematic Reviews of Interventions.^[47]^ Random sequence generation, allocation concealment, the use of blindness, integrity of the outcome data, selective outcome reporting, and other risks of bias will be assessed by 2 reviewers (YZ and HW). According to the above 6 items, each study will be assessed as “high risk,” “low risk,” or “unclear risk.” For unclear items in the study, contact the corresponding author for details. Any disagreement will be resolved by discussion with a third reviewer (J-jF).

### 2.6 Statistical analysis

#### 2.6.1 Meta-analysis

Meta-analysis will be performed using Review Manager 5.3, a statistical software provided by the Cochrane collaboration. Continuous data are presented as mean difference (MD) with 95% confidence interval (CI) and *P* value. Count data are presented as relative risk ratio (RR) with 95% CI and *P* value. *P* < .05 will be considered statistical significance for all analyses. The potential heterogeneity will be manifested by the *χ*^2^ test and *I*^2^ test.

#### 2.6.2 Dealing with missing data protocol

If missing data are detected, reviewers (LL and E-qQ) will contact the original study investigators to obtain the missing data. For the missing data that cannot be obtained, an intention-to-treat analysis will be performed if possible and a sensitivity analysis will be conducted.^[48]^

#### 2.6.3 Assessment of heterogeneity

A standard *χ*^2^ test with a significance level of *P* < .1 will be used for testing statistical heterogeneity. When *I*^2^ value is <50%, the study will not be considered to have heterogeneous.

#### 2.6.4 Publication bias

If >10 studies are included, funnel plots will be used to detect potential publication bias. The Egger regression test will be used to determine the asymmetry of the funnel plot.^[49]^

#### 2.6.5 Data synthesis

When the heterogeneity test indicated the existence of heterogeneity between studies (*P* ≤ .05, *I*^2^ ≥ 50%), the random-effect model was used for combined analysis, or subgroup analysis or sensitivity analysis was conducted according to the heterogeneity source. When the heterogeneity test indicated that there was no heterogeneity between the groups (*P* > .05, *I*^2^ < 50%), the fixed-effect model was used for combined analysis. If quantitative synthesis is not appropriate, qualitative analysis will be carried out.

#### 2.6.6 Subgroup analysis and investigation of heterogeneity

Subgroup analyses will be performed to explain heterogeneity if possible. Factors such as different CHM dosage forms, duration of treatment, measurements of results will be considered.

#### 2.6.7 Sensitivity analysis

When the heterogeneity is significantly different from the methodological quality of the included studies, the stability of the results can be assessed by sensitivity analysis. The effect of methodological quality, sample size, and missing data will be assessed. In addition, the analysis will be repeated after excluding low-methodological quality studies.

#### 2.6.8 Quality of evidence

The quality of evidence for the main outcomes will be assessed with the GRADE (the Grading of Recommendations Assessment, Development and Evaluation) approach.^[50]^ These 5 items will be investigated, including limitations of study design, inconsistencies, inaccuracies, indirection, and publication bias. There are 4 levels of assessment: high, medium, low, and extra-low quality.

## 3 Discussion

The therapeutic goals for stable COPD are to relieve clinical symptoms and to prevent acute exacerbations.^[1]^ However, increasing evidence show that patients still have cough, sputum production, dyspnea, or other symptoms, and acute exacerbation still occurs frequently as well as high mortality despite the use of drugs recommended by clinical guidelines.^[51]^ Studies have also shown that the long-term use of bronchodilators will increase the risk of arrhythmia,^[52,53]^ and inhaled glucocorticoids (ICS) increases the risk of pneumonia and diabetes.^[54,55]^ Discovery of other therapeutic strategy or medications for COPD is in urgent need. CHM, as an alternative and complementary therapy for western medicine, has been widely used in the treatment of stable COPD. Evidence has shown that compared with western medicine treatment alone, CHM with western medicine treatment reduced the risk of exacerbation of COPD and improved quality of life.^[22,30]^

RCTs are the best way to study the safety and efficacy of new treatments. Placebo is a substance that has neither medical effect nor adverse reactions, but whose dosage form, size, color, weight, taste, and smell are similar to those of therapeutic drugs.^[56]^ Placebo control in RCTs is an important method to ensure the implementation of blind method. Setting placebo-controlled can eliminate the bias caused by subjective factors of researchers and subjects, eliminate the influence of natural progression of diseases, distinguish the real adverse reactions caused by test drugs, and can directly evaluate the difference between test drugs and placebos under test conditions to determine whether the test drugs are really effective.^[57,58]^ It is believed that placebo control has incomparable advantages in the accuracy of research results.^[59]^

Therefore, this systematic review will provide a detailed summary of the efficacy and safety of CHM compared to placebo in the treatment of COPD based on current evidence, to evaluate the real clinical efficacy of CHM. The strength of this study is the inclusion of a wide range of outcomes that will be helpful for comprehensively evaluating the efficacy and safety of CHM in the treatment of COPD. Additionally, this study will be carried out by several reviewers in strict accordance with PRISMA guidelines to ensure the quality of the study. This study also has some limitations, such as potential unpublished studies may introduce certain bias, and difference in the forms of CHM and duration of therapy may cause heterogeneity. We will conduct subgroup analysis to resolve these questions. The study conclusions will help to provide robust evidence for the application of CHM for respiratory physicians, health policymakers, and patients with COPD.

## 4 Ethics and dissemination

No ethical approval is required because the study is based on published data. The results of this systematic review will be disseminated through peer-reviewed publications.

## Author contributions

**Conceptualization:** Chan Xiong, Hua Wei.

**Data curation:** Chan Xiong, Yu Li.

**Formal analysis:** Chan Xiong, Guang-tong Zhuang.

**Project administration:** Yan Zeng, Li-hong Zhao.

**Resources:** Juan-juan Fu.

**Software:** Chan Xiong, Chen-yi Li.

**Visualization:** Lin Li, Er-qi Qin.

**Writing – original draft:** Chan Xiong, Yu Li.

**Writing – review & editing:** Juan-juan Fu.
